# *Toll like receptor 4* gene Asp299Gly polymorphism increases the risk of diabetic microvascular complications: a meta analysis

**DOI:** 10.1186/s13098-022-00849-2

**Published:** 2022-06-07

**Authors:** Yuqi Zhang, Huanhuan Li, Chenyi Wang, Haihong Lv, Songbo Fu

**Affiliations:** 1 Department of Endocrinology, The First Hospital of Lanzhou University, No. 1 Donggang West Road, Lanzhou, 730000 Gansu People’s Republic of China; 2 The First Clinical Medical College of Lanzhou University, Lanzhou, 730000 Gansu People’s Republic of China

**Keywords:** Toll like receptor 4 (*TLR4*), Asp299Gly(rs4986790, 896A > G), Gene polymorphism, Diabetic microvascular complications (DMI), Meta analysis

## Abstract

**Objective:**

The relationship between *Toll like receptor 4(TLR4)* gene Asp299Gly polymorphism and diabetic microvascular complications (DMI) is unclear. Therefore, the aim of this meta analysis was to explore the relationship between *TLR4* Asp299Gly polymorphism and DMI.

**Methods:**

System search PubMed, Web of science, Springer, Cochrane library, ELSEVIER, Wanfang database, VIP, CNKI, a case–control study of the correlation between *TLR4* gene Asp299Gly polymorphism and DMI published before June 2020 was collected.

**Results:**

We included 6 articles, a total of 11 studies involving patients with type 2 diabetes mellitus (T2DM) complicated by microvascular complications 1834 cases, without corresponding microvascular complications 4069 cases. *TLR4* gene Asp299Gly polymorphism increased the risk of microvascular complications in T2DM (dominant model OR = 1.52, 95% CI 1.10–2.09, p = 0.01; allelic model OR = 1.42, 95% CI 1.02–1.96, p = 0.04). Subgroup analysis by race and different type of microvascular complications, we found that *TLR4* gene Asp299Gly polymorphism was associated with increased risk of microvascular complications in the Caucasian population (dominant model OR = 1.69, 95% CI 1.22–2.35, P = 0.002; allelic model OR = 1.56, 95% CI 1.10–2.21, P = 0.01) and increased the risk of retinopathy in patients with T2DM(dominant model OR = 1.81, 95% CI 1.04–3.14, P = 0.03; allelic model OR = 1.77, 95% CI 1.05–2.98, P = 0.03).

**Conclusion:**

*TLR4* gene Asp299Gly polymorphism was associated with increased risk of microvascular complications in patients with T2DM, especially diabetic retinopathy (DR).

## Introduction

Diabetic microvascular complications (DMI) are the most common chronic complications of diabetes, which are the main causes of disability and death in diabetic patients [1], including diabetic retinopathy, diabetic nephropathy and diabetic neuropathy [2]. Many risk factors play an important role in the onset of DMI, such as course of diabetes, blood glucose control level, BMI and age [3, 4]. However, DMI are a complex metabolic disease composed of multiple factors including genetic and environmental factors, its specific pathogenesis is still unclear, causing difficult in diagnosis and treatment. Due to the high morbidity and mortality of DMI, early diagnosis and treatment are particularly important. Recent studies have shown that some macromolecules such as homocysteine (HCY), troponin T (TnT) and asymmetric dimethylarginine (ADMA) play an important part in the pathogenesis of diabetic vascular diseases, which have good clinical diagnostic value and can be considered as diagnostic markers for diabetic vascular complications [5–7]. These macromolecules are more widely used in diagnosis of diabetic macrovascular complications, however, are less in DMI. Therefore, the discovery of biomarkers for the diagnosis of DMI requires further investigation. A cross-sectional retrospective study involving 90 patients showed that the expression of TLR4 in Diabetic polyneuropathy (DPN) patients was significantly higher than that in T2DM patients and healthy controls, and the up-regulation of TLR4 level would significantly increase the risk of DPN, which suggest that TLR4 may be a potential and sensitive diagnostic biomarker for diabetic neuropathy [8].

*TLR4* gene, encodes TLR4 protein, located on chromosome 9Q32-33 and its variations can alter TLR4's recognition and interaction functions [9]. TLR4 is considered a key member of the TLRs families, as a highly conserved pattern recognition receptor, which can bind to specific ligands that induces activation of nuclear factor-κB, leading to an increase in downstream proinflammatory cytokines and adhesion molecules, involved in the occurrence of inflammatory responses by MyD88 dependent or non-dependent signaling pathways [10, 11]. The polymorphism of *TLR4*Asp299Gly gene may affect ligand binding, folding efficiency, cell surface expression and protein stability, while the polymorphism of Thr399Ile gene has little effect [12].

Recently, studies on the relationship between *TLR4* gene polymorphism and type 2 diabetes mellitus (T2DM) and its complications have attracted extensive attention. Yin et al. indicated that *TLR4* gene Asp299Gly and Thr399Ile polymorphisms are not associated with increased T2DM risk through a meta-analysis [13], whereas Chang et al. pointed out some problems that still exist or need to be improved in the meta-analysis of Yin et al. [14]. Several studies have shown that *TLR4* gene polymorphism is associated with the occurrence of DMI, among which the research on *TLR4*Asp299Gly is the hotspot [15–24]. Khaghanzadeh et al. [15] showed that c.1196C > T and 896A > G variants might increase the risk of DR by involving in the dysregulation of serum lipid levels and hyperglycemia. Buraczynska et al. [16] concluded that the G(rs4986790) polymorphism of *TLR4*Asp299Gly allele increased the risk of DR. That is to say, a significant increase of G allele frequency was observed in DR compared to those without it in T2DM (OR = 2.12, 95% CI (1.43–3.12), p = 0.0002). Yet Balistreri et al. [17] revealed that there was no correlation between rs4986790 gene polymorphism and the occurrence of DR and diabetic neuropathy. In view of the conclusions on the correlation between p.Asp299Gly and DMI are not consistent, so we performed this meta analysis to further study the relationship between them, to determine whether *TLR4* gene polymorphism can be considered as a risk factor or diagnostic marker for DMI.

## Materials and methods

### Literature retrieval methods

The electronic databases PubMed, Web of science, Springer link, Cochrane library, ELSEVIER, Wanfang database, VIP, CNKI were searched using the following terms: “(TLR4 or toll-like receptor-4) and (gene or polymorphism or allele or genotype or variant or variation or mutation) and (diabetic or diabetes) and (nephropathy or retinopathy or neuropathy)”. All literature is limited to Chinese or English published before June 20 2020 ranging from 2000 to 2020 (last research was updated on May 2020). At the same time, the literature was traced, and the literature related to this study was searched according to the abstract and the full text was obtained.

### Inclusion and exclusion criteria

Inclusion criteria: (1) study of the relationship between *TLR4*Asp299Gly gene polymorphism and diabetic microvascular complications(including diabetic retinopathy, diabetic nephropathy and diabetic neuropathy); (2) case–control study; (3) population studies; (4) the original text provides the frequency of the genotype or allele or can be extrapolated; (5) the control group was type 2 diabetes patients without corresponding microvascular complications; (6) sufficient data are available to estimate OR values and 95 percent confidence intervals; (7) the control group met the law of genetic balance. Exclusion criteria: (1) critical literature, case reports, conference abstracts, letters etc.; (2) non-case–control studies; (3) the original text does not provide raw data or data is insufficient.

### Data extraction

All the data are extracted independently by the two authors according to the set retrieval strategy and the inclusion criteria, the dissenting literature was judged by the third author and finally reached an agreement. The data extracted in the literature included the first author, year of publication, country, race, type of diabetic microvascular complications, sample size of case group and control group, distribution of genotypes and alleles. In addition, whether the control group included in the study complied with Hardy–Weinberg balance (HWE) was also mentioned in this paper.

### Quality evaluation

The literature quality assessment scale of case–control studies, namely Newcastle-Ottawascale (NOS), was used for evaluation, including the selection of study subjects, comparability between groups and measurement of exposure factors. The scores ranged from 0 to 9, and the higher the score, the better the quality of the literature.

### Statistical analysis

Genetic balance test was carried out for each genotype distribution in the control group. The HWE P < 0.05 was considered significant. Review Manager 5.3 statistical software was used to analyze different models of *TLR4*Asp299Gly loci(dominant model, allelic model, recessive model and additive model) in case group and control group. That is, the relationship between *TLR4*Asp299Gly gene polymorphism and diabetic microvascular complications was estimated by ORs and its 95% confidence interval under different genetic models: dominant model(G/G + A/G vs A/A), allelic model(G vs A), recessive model(G/G vs A/G + A/A), additive model(G/G vs A/A). Judging the heterogeneity by I^2^ and P, if the heterogeneity is slight (P ≥ 0.1, I^2^ < 50), the fixed effect model was used. When there is obvious heterogeneity (P < 0.1, I^2^ > 50%), the possible source of heterogeneity is subgroup analysis or sensitivity analysis, and heterogeneity is eliminated as far as possible. If the reason of heterogeneity is not found, random effect model is used for combined statistical analysis. The final result was statistically significant with P < 0.05.

## Results

### Study characteristics

**Fig. 1:**
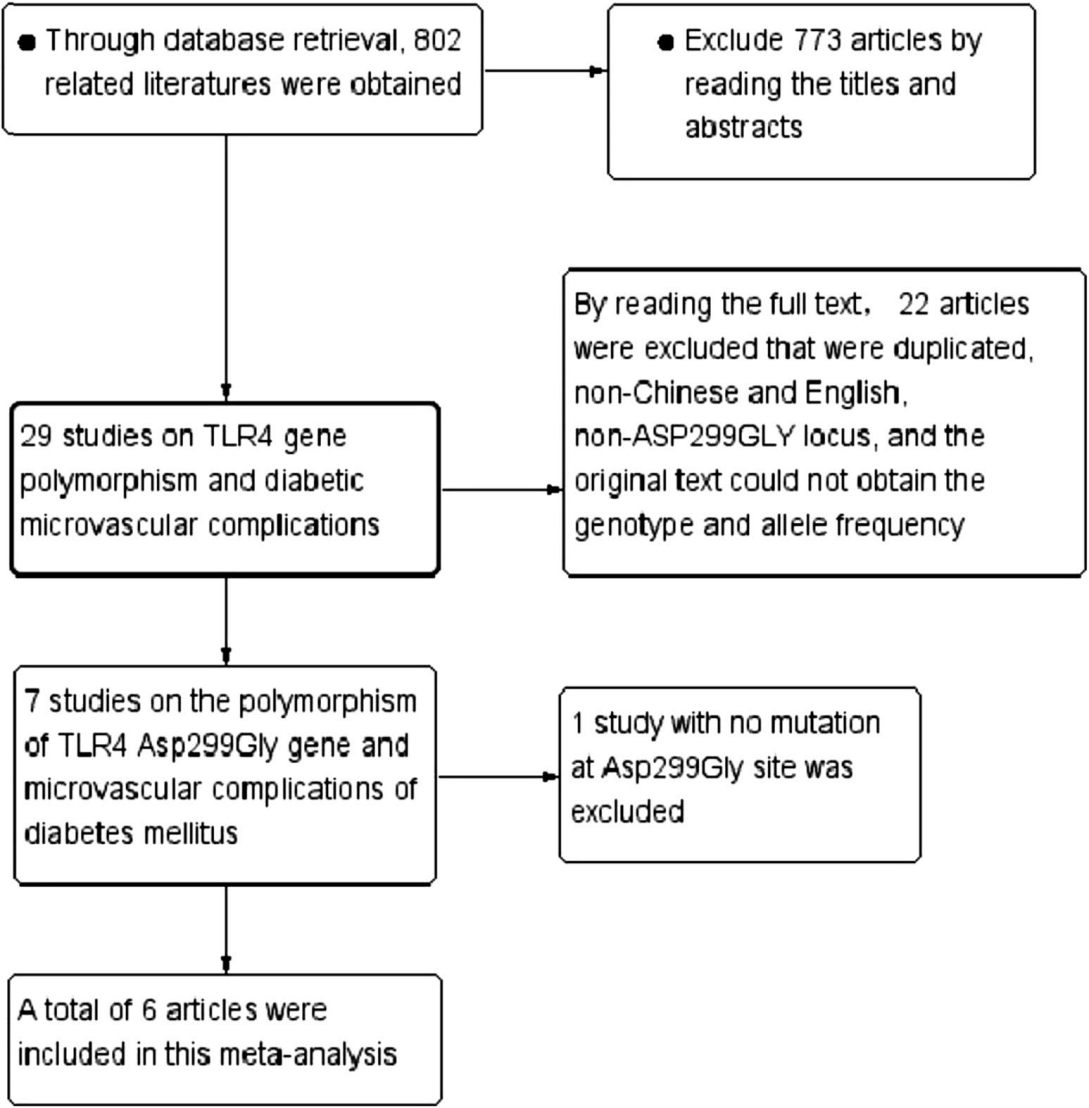
The flow chart of literature search and study selection

The study selection process is detailed in Fig. 1. According to the searching strategy, 802 potentially eligible articles were identified in our initial search. We reviewed the titles and abstracts, 773 articles were excluded. By reading the full text to exclude duplicate, non-English, non-Asp299Gly locus, original text cannot obtain genotype and allele frequency excluded 22, 7 articles were initially included [15–20, 24]. Through data collation, 1 literature with no mutation in Asp299Gly site was not included in this meta analysis [24]. Finally, 6 articles that met the inclusion criteria were included in this study [15–20], including 11 case–control studies. The main characteristics of the selected studies are listed in Table 1, with a total of 11 studies of 1834 cases and 4069 controls for investigating the association between DMI and *TLR4*Asp299Gly polymorphism. Ethnic groups among these studies were as follows: 2 were Asians involving 478 population and 4 were Caucasians with 5425 population.

**Table 1:** Characteristics of studies included in this meta-analysis

Author	Year	Country	Ethnicity	Language	Case	Control	Research method	Score
Buraczynska [16]	2016	Poland	Caucasian	English	368	722	RFLP-PCR	9
Balistreri [17]	2014	Italy	Caucasian	English	48	319	RFLP-PCR	9
Khaghanzadeh [15]	2020	Iran	Asian	English	11	89	SSP-PCR	8
Balistreri* [17]	2014	Italy	Caucasian	English	112	255	RFLP-PCR	9
Buraczynska* [16]	2016	Poland	Caucasian	English	342	748	RFLP-PCR	9
Buraczynska* [18]	2009	Poland	Caucasian	English	352	512	RFLP-PCR	9
Singh* [19]	2014	India	Asian	English	128	250	RFLP-PCR	9
Zaharieva * [20]	2017	Bulgaria	Caucasian	English	10	75	PCR	8
Balistreri** [17]	2014	Italy	Caucasian	English	74	293	RFLP-PCR	9
Buraczynska**[16]	2016	Poland	Caucasian	English	302	788	RFLP-PCR	9
Zaharieva** [20]	2017	Bulgaria	Caucasian	English	87	18	PCR	8

*Diabetic retinopathy; **diabetic neuropathy; without*diabetic nephropathy. PCR: Polymerase chain reaction; SSP-PCR: Polymerase chain reaction sequence specific primers; PCR–RFLP: PCR-restriction fragment length polymorphism

NOS score results showed that the overall quality of the literature included in the study was high. The distribution of genotypes and alleles in the case group and the control group and the results of genetic balance were shown in Table 2, indicating that the studies included are in accordance with the law of genetic balance.

**Table 2:** *TLR4*Asp299Gly distribution of genotypes and alleles

Author	Year	Genotype (case/control)			Allele (case/control)		HWE
		AA	AG	GG	A	G	(Y/N)
Balistreri [17]	2014	43/293	5/26	0/0	91/612	5/26	Y
Buraczynska [16]	2016	329/658	37/61	2/3	695/1377	41/67	Y
Khaghanzadeh [15]	2020	10/71	1/18	0/0	21/160	1/18	Y
Balistreri* [17]	2014	102/234	10/21	0/0	214/489	10/21	Y
Buraczynska* [16]	2016	292/695	48/50	2/3	632/1440	52/56	Y
Buraczynska* [18]	2009	309/487	40/24	3/1	658/998	6/26	Y
Singh* [19]	2014	99/188	28/61	1/1	226/437	30/63	Y
Zaharieva* [20]	2017	7/7	3/4	0/0	17/146	3/4	Y
Balistreri** [17]	2014	63/273	11/20	0/0	137/566	11/20	Y
Buraczynska** [16]	2016	273/714	27/71	2/3	573/1499	31/77	Y
Zaharieva** [20]	2017	80/17	7/1	0/0	167/35	7/1	Y

*Diabetic retinopathy; **diabetic neuropathy; without*diabetic nephropathy; AA: AA genotype; AG: AG genotype; GG: GG genotype; A: A allele; G: G allele; HWE: Hardy–Weinberg equilibrium; Y: Yes; N: No

### Meta-analysis results

#### The relationship between *TLR4*Asp299Gly gene polymorphism and DMI

**Fig. 2:**
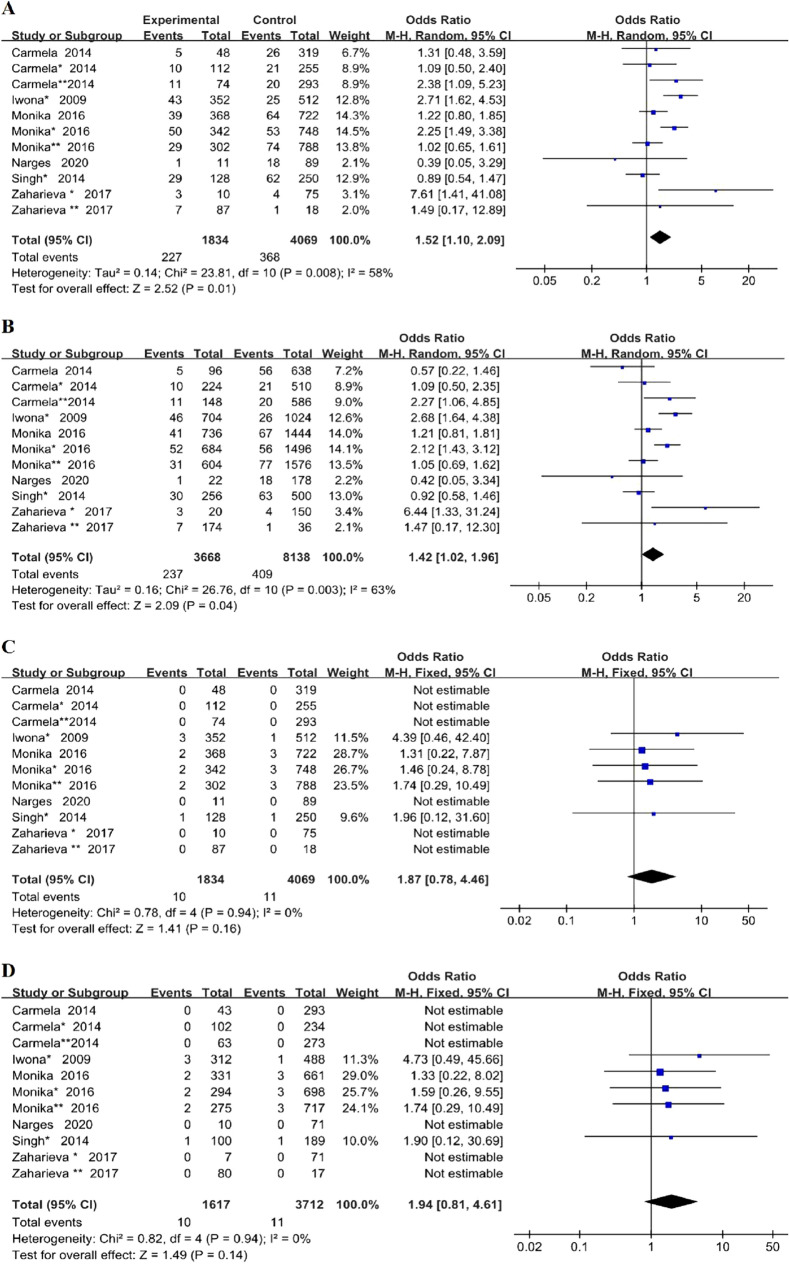
Forest plots of the meta-analysis for *TLR4*Asp299Gly gene polymorphism associated with DMI in different genetic model. **A** Dominant model: G/G + A/G vs A/A; **B** allelic model: G allele vs A allele; **C** recessive model: G/G vs A/G + A/A; **D** additive model: G/G vs A/A

Heterogeneity is significant among included studies in the dominant model and allelic model (P = 0.008, I^2^ = 58%; P = 0.003, I^2^ = 63% respectively), but not in the recessive model and additive model (P = 0.94, I^2^ = 0%; P = 0.94, I^2^ = 0% respectively). So we chose the random effects model while we analyze the dominant model and the allele model and used fixed-effects model to analyze the data of recessive model and additive model, as shown in Fig. 2 (A: dominant model, B: allele model, C: recessive model, D: additive model).

As shown in Fig. 2, our meta-analysis showed that *TLR4*Asp299Gly gene polymorphism was associated with increased risk of DMI under dominant model (OR = 1.52, 95% CI (1.10–2.09), p = 0.01) and allelic model (OR = 1.42, 95% CI (1.02–1.96), p = 0.04). However, there was no significant correlation between this gene polymorphism and DMI either using a recessive model (OR = 1.87, 95% CI (0.78–4.46), p = 0.16) or additive model (OR = 1.94, 95% CI (0.81–4.61), p = 0.14).

#### Subgroup analysis

**Fig. 3:**
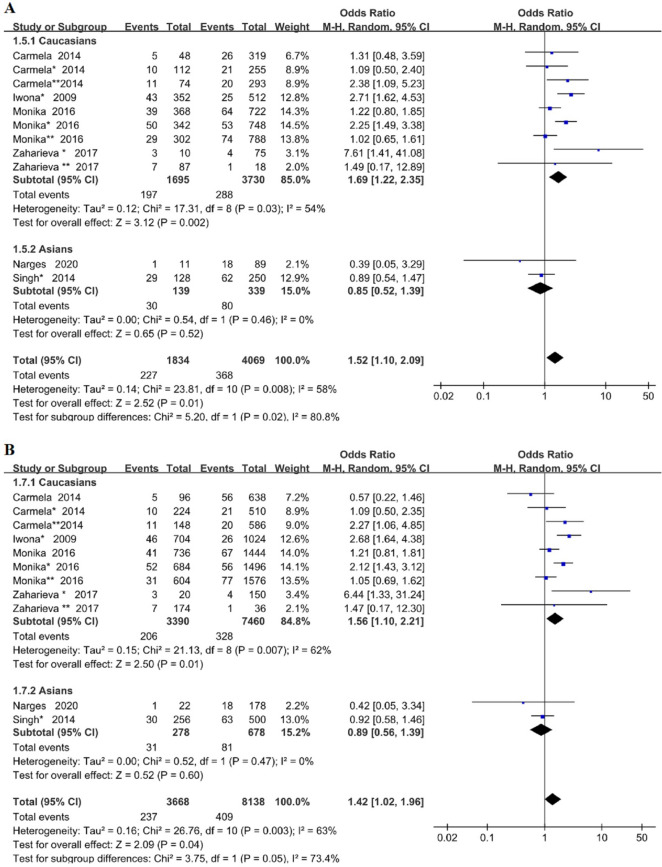
Forest plots of the meta-analysis for *TLR4*Asp299Gly gene polymorphism associated with DMI in different genetic model after stratification analysis by ethnicity. **A** Dominant model: G/G + A/G vs A/A; **B** allelic model: G allele vs A allele

Subgroup analysis was performed by different ethnic groups, we can draw the conclusion that *TLR4*Asp299Gly gene polymorphism increased the risk of microvascular complications in Caucasian patients with T2DM (dominant model OR = 1.69, 95% CI (1.22–2.35), P = 0.002; allelic model OR = 1.56, 95% CI (1.10–2.21), P = 0.01). However, we found no correlation in the Asian population (dominant model OR = 0.85, 95% CI (0.52–1.39), P = 0.52; allelic model OR = 0.89, 95% CI (0.56–1.39), P = 0.60), as shown in Fig. 3 (A: dominant model; B: allele model). This study showed that there were racial differences in *TLR4*Asp299Gly gene polymorphism.

**Fig. 4:**
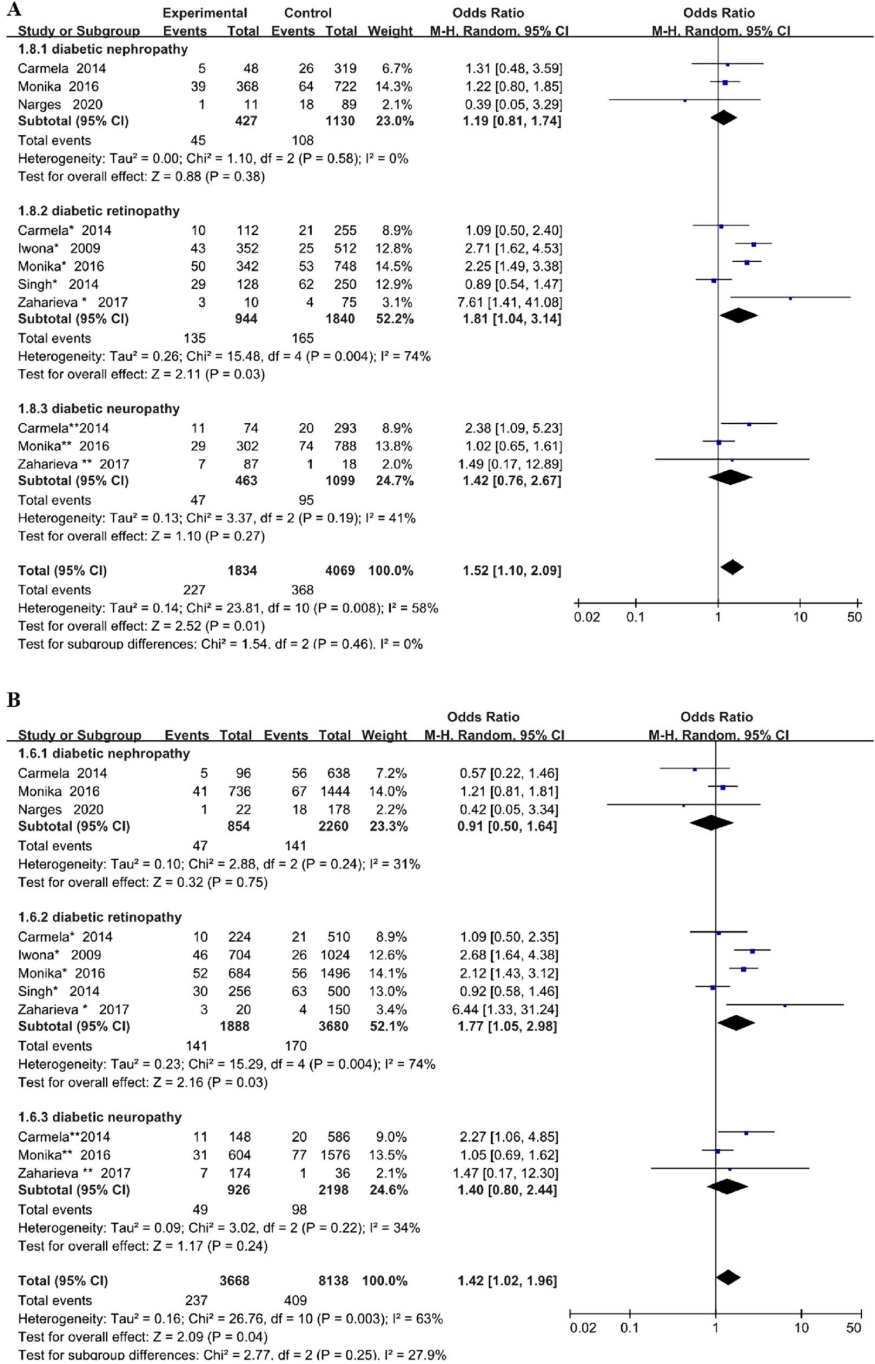
Forest plots of the meta-analysis for *TLR4*Asp299Gly gene polymorphism associated with DMI after stratification analysis by different types of microvascular complications in different genetic model. **A** Dominant model: G/G + A/G vs A/A; **B** allelic model: G allele vs A allele

With regard to the specific type of DMI, we also conducted the subgroup analysis stratified by DR or DN or diabetic neuropathy. The results showed that *TLR4*Asp299Gly genetic polymorphism increased the risk of retinopathy in T2DM under dominant model and allelic model (OR = 1.81, 95% CI (1.04–3.14), P = 0.03; OR = 1.77, 95% CI (1.05–2.98), P = 0.03 respectively). But no significant association was found between DN or diabetic neuropathy and this gene polymorphism (DN: dominant model OR = 1.19, 95% CI (0.81–1.74), P = 0.38; allelic model OR = 0.91, 95% CI (0.50–1.64), P = 0.75; diabetic neuropathy: dominant model OR = 1.42, 95% CI (0.76–2.67), P = 0.27; allelic model OR = 1.40, 95% CI (0.80–2.44), P = 0.24), as shown in Fig. 4 (A: dominant model, B: allele model). Meta-analysis of the associations of *TLR4*Asp299Gly polymorphism with DMI risk was shown in Table 3, in which we include subgroups, test of association, test of heterogeinity.

**Table 3:** Meta-analysis of the associations of *TLR4*Asp299Gly polymorphism with DMI risk

Genetic model	Comparison	Test of association		Test of heterogeneity	
		OR (95% CI)	P-value	P	I^2^ (%)
Dominant model	G/G + A/G vs A/A	1.52 (1.10–2.09)	**0.01**	0.008	58.0
Allelic model	G vs A	1.42 (1.02–1.96)	**0.04**	0.003	63.0
Recessive model	G/G vs A/G + A/A	1.87 (0.78–4.46)	0.16	0.94	0.0
Additive model	G/G vs A/A	1.94 (0.81–4.61)	0.14	0.94	0.0
Subgroups					
Ethnicity					
Caucasians	G/G + A/G vs A/A	1.69 (1.22–2.35)	**0.002**	0.03	54.0
	G vs A	1.58 (1.10–2.21)	**0.01**	0.007	62.0
Asians	G/G + A/G vs A/A	0.85 (0.52–1.39)	0.52	0.46	0.0
	G vs A	0.89 (0.56–1.39)	0.60	0.47	0.0
Difference of DMI types					
Diabetic retinopathy	G/G + A/G vs A/A	1.81 (1.04–3.14)	**0.03**	0.004	74.0
	G vs A	1.77 (1.05–2.98)	**0.03**	0.004	74.0
Diabetic nephropathy	G/G + A/G vs A/A	1.19 (0.81–1.74)	0.38	0.58	0.0
	G vs A	0.91 (0.50–1.64)	0.75	0.24	31.0
Diabetic neuropathy	G/G + A/G vs A/A	1.42 (0.76–2.67)	0.27	0.19	41.0
	G vs A	1.40 (0.80–2.44)	0.24	0.22	34.0

Bold values indicate P-value < 0.05, that is, the combined effect size is statistically significant

#### Sensitivity analysis

The method of one-by-one exclusion was used to conduct sensitivity analysis, and the results showed that the combined OR value and 95% CI were not significantly affected in the whole population under the dominant model, indicating that the meta results were stable and reliable under the analysis of this model. And under the allelic model analysis one by one to eliminate are included in each of the study results show that OR value and 95% CI had essential changes after the merger, so we have to cut out the significant change in merger results in 5 cases of study (Carmela * * 2014, Iwona *2009, Monika2016, Monika * 2016, Zaharieva * 2017), the merge OR = 0.95, 95% CI (0.72, 1.25), P = 0.70, heterogeneity, P = 0.81, I^2^ = 0. We found significant changes in OR value and 95%CI, and the heterogeneity disappeared, suggesting that the heterogeneity may be caused by the excluded studies.

#### Publication bias

**Fig. 5:**
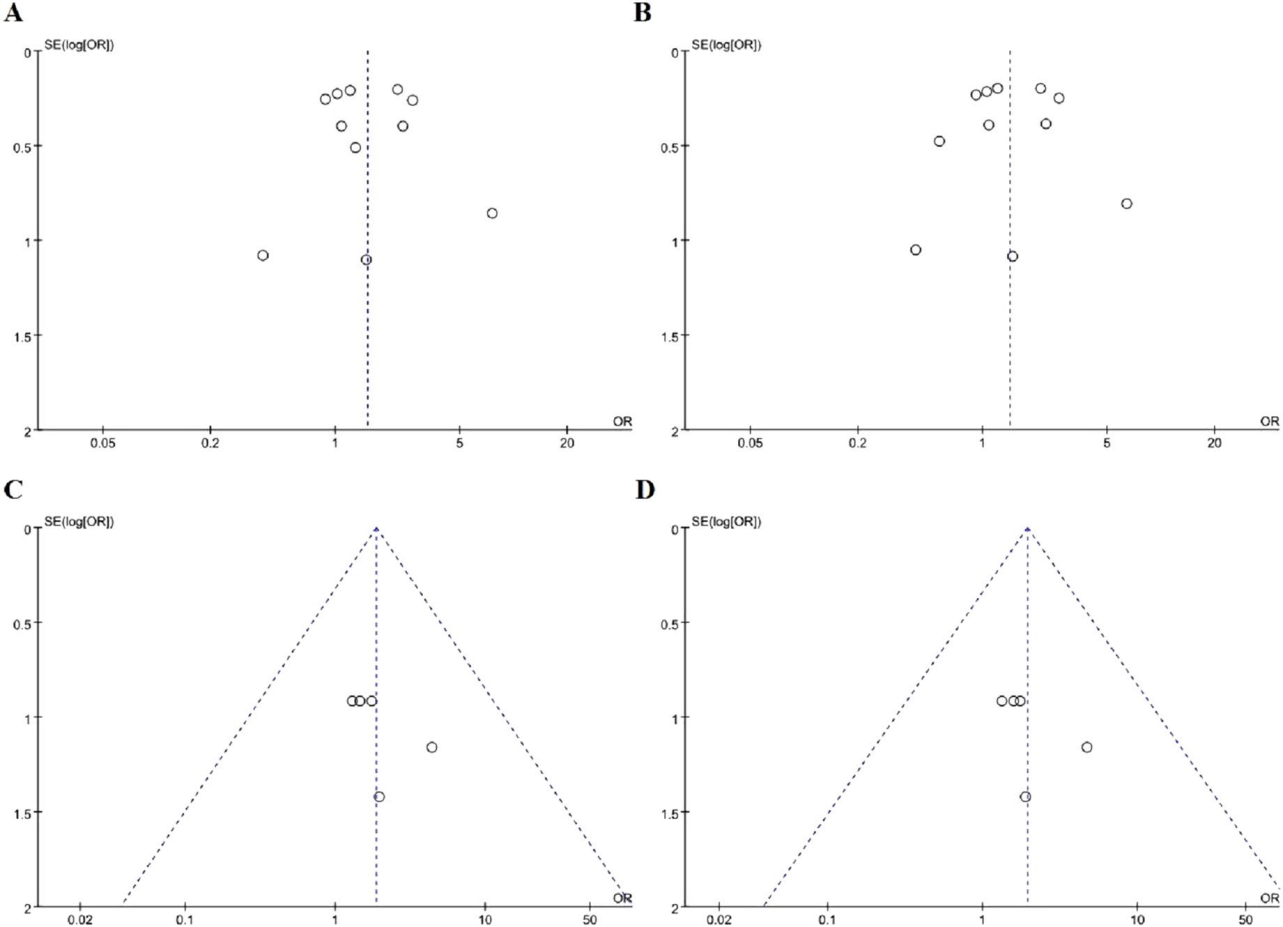
Funnel plots for *TLR4*Asp299Gly gene polymorphism and DMI risk in different genetic model. **A** Dominant model: G/G + A/G vs A/A); **B** allelic model: G allele vs A allele; **C** recessive model: G/G vs A/G + A/A; **D** additive model: G/G vs A/A

**Fig. 6:**
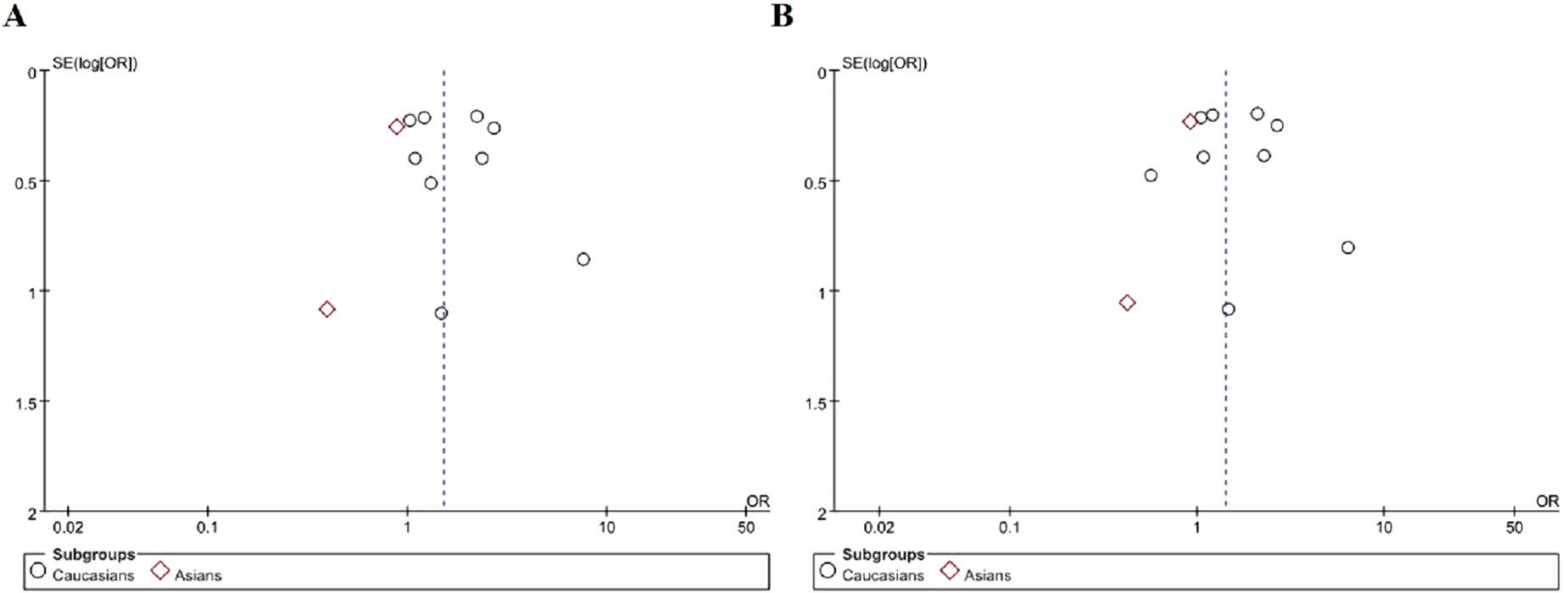
Funnel plots for *TLR4*Asp299Gly gene polymorphism and DMI risk in different genetic model after stratification analysis by ethnicity. **A** Dominant model: G/G + A/G vs A/A; **B** allelic model: G allele vs A allele

**Fig. 7:**
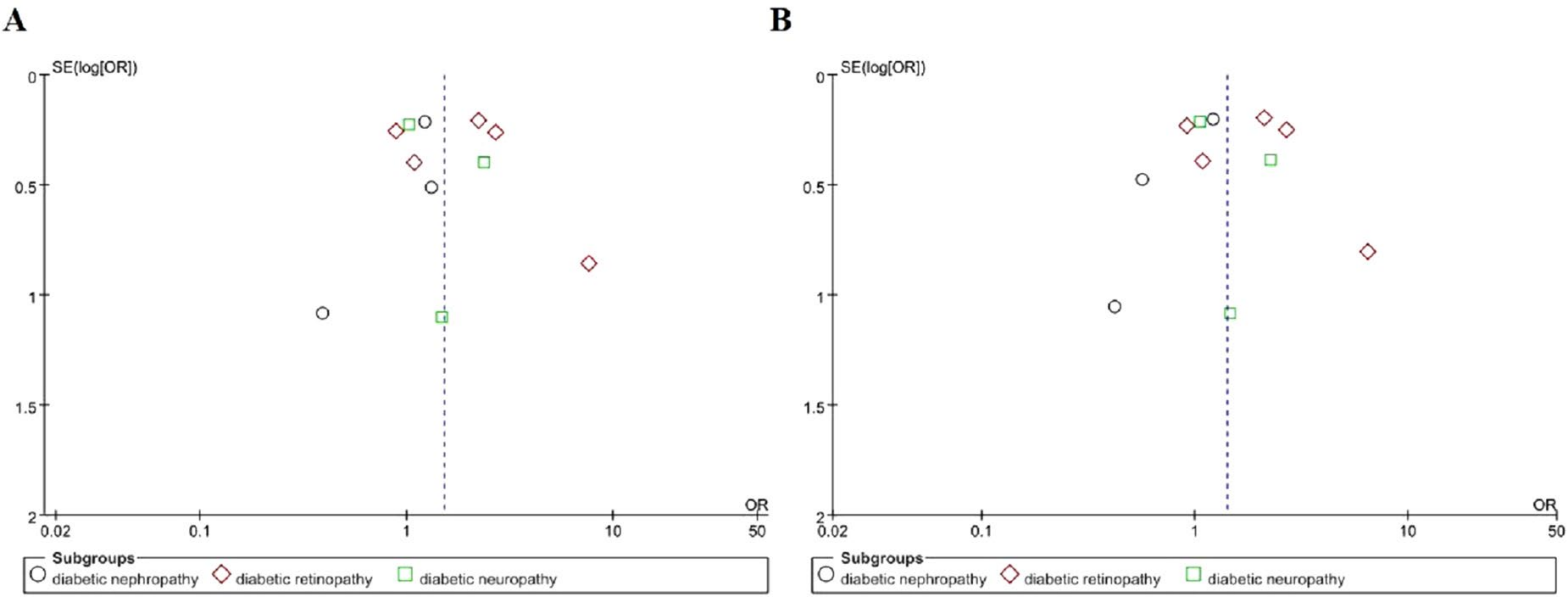
Funnel plots for *TLR4*Asp299Gly gene polymorphism and DMI risk in different genetic model after stratification analysis by specific type of DMI. **A** Dominant model: G/G + A/G vs A/A; **B** allelic model: G allele vs A allele

Funnel plots was used to evaluate publication bias among included studies. The included studies in the funnel plots are basically symmetrical around the center line, suggesting that there was no significant publication bias among included studies, as shown in Figs. 5, 6 and 7.

## Discussion

TLR4, as one of the key members of the earliest and most clearly studied Toll-like Receptors (TLRs) family, consists of extracellular domain, transmembrane domain and intracellular domain. The extracellular domain of 22 leucine-rich repeats mediates Lipopolysaccharide (LPS) recognition and receptor dimerization, while the intracellular domain is structurally similar to interleukin 1(TIR1) and plays an important role in downstream signal transduction [25]. TLR4 is mainly distributed on the surface of B lymphocytes, monocytes/macrophages, smooth muscle cells, dendritic cells and other cells [26]. As a highly conserved pattern recognition receptor, it can interact with endogenous ligands such as Heat shock protein(HSP), fibrinogen, High Mobility Group Box 1 protein(HMGB1), Oxidized low density lipoprotein(OX-LDL) and exogenous ligands such as LPS released by Gram-negative bacteria [27–29]. The activation of Nuclear factor-kappa B(NF-κB) was induced by regulating the dependent or independent signaling pathways of Myeloid differentiation factor 88(MyD88) [30]. TLR4/MyD88/NF-κB signaling pathway leads to the increase of downstream pro-inflammatory cytokines and adhesion molecules, participate in the occurrence of inflammatory reactions [31], and cause the pathological changes such as vascular extravasation or increased vascular permeability, vascular obstruction, and degeneration and pathological changes of neovascularization, thus leading to the occurrence and development of DMI [32, 33]. Activation of the TLR4 signaling pathway mediates the inflammatory response, which is associated with *TLR4* gene polymorphism [34]. Genetic variation of *TLR4* can alter TLR4’s recognition and interaction functions, and then, change the immune response [9]. It has been reported that the replacement of the conserved Asp with Gly at position 299 theoretically cause disruption of the alpha helix protein structure, leading to extended beta strain, which is less functional [35]. As one of the most common variants of *TLR4*, Asp299Gly gene polymorphism is known to alter the extracellular domain of the receptor, resulting in hyporesponsiveness to LPS, attenuating the TLR4 signaling pathway and reducing the inflammatory response to Gram-negative pathogens [36, 37]. In addition, Zaharieva et al. also noted that it is mainly Asp299Gly rather than Thr399Ile polymorphism that leads to peptide structural modifications, changes in ligand-receptor interactions, post-receptor signal transduction, and further cytokine production [18].

Several studies have shown that *TLR4* gene polymorphism is closely related to the occurrence and development of DMI [15–24]. Multiple loci of the *TLR4* gene polymorphisms such as rs4986791, rs1927911, rs1927914, rs10759931, are associated with DMI. What’s more, the relationship between *TLR4*Asp299Gly gene polymorphism and DMI is a hot topic [15–21, 23]. The mutant of *TLR4*Asp299Gly/Thr399Ile will change the structure of TLR4 itself, leading to problems in ligand binding. This structural/functional irregularity seems ultimately to lead to a blunt immune response, such as the reduction of IgA production against microbial targets and the decrease of functional TLR4 levels [38]. The results of our meta analysis covering a total of 1834 cases and 4069 controls showed that *TLR4*Asp299Gly gene polymorphism increased the risk of DMI under dominant model and allele model (OR = 1.52, 95% CI (1.10–2.09), p = 0.01; OR = 1.42, 95% CI (1.02–1.96), p = 0.04 respectively). However, we found that there was no correlation between them under the recessive model and additive model (OR = 1.87, 95% CI (0.78–4.46), p = 0.16; OR = 1.94, 95% CI (0.81–4.61), p = 0.14 respectively). The conclusion of Carmela, CS Aioanei and Gottfried were contradictory to our results, which may be attributed to the ethnic difference of the included population and the difference of DMI types [17, 21, 23].

So it is necessary to perform subgroup analysis, we found that the significant association was observed in DR subgroup under dominant model and allele model (OR = 1.81, 95% CI (1.04–3.14), P = 0.03; OR = 1.77, 95% CI (1.05–2.98), P = 0.03 respectively) from subgroup analysis of the difference of DMI types. Nevertheless, no significant association was observed between *TLR4*Asp299Gly gene polymorphism and DN and diabetic neuropathy subgroup (p > 0.05). The studies of Buraczynska et al. [16], Buraczynska et al. [18], and Zaharieva [20] were consistent with our results, which further confirmed that *TLR4*Asp299Gly gene polymorphism was significantly associated with increased risk of DR. Therefore, we can infer that although DR, DN and diabetic neuropathy were included in microvascular complications, there still exists some differences in the pathogenesis.

To our knowledge, this is the first meta-analysis to investigate the relationship between *TLR4*Asp299Gly gene polymorphism and DMI. The results showed that *TLR4*Asp299Gly gene polymorphism increased the risk of retinopathy in patients with T2DM(dominant model OR = 1.81, 95% CI (1.04–3.14), P = 0.03; allelic model OR = 1.77, 95% CI (1.05–2.98), P = 0.03), but there was no correlation between this gene polymorphism and DN or diabetic neuropathy(DN: dominant model OR = 1.19, 95% CI (0.81–1.74), P = 0.38; allelic model OR = 0.91, 95% CI (0.50–1.64), P = 0.75; diabetic neuropathy: dominant model OR = 1.42, 95% CI (0.76–2.67), P = 0.27; allelic model OR = 1.40, 95% CI (0.80–2.44), P = 0.24). Hence, we speculated that the expression of TLR4 or its ligands in retinal vessels, glomerular microvessels and neural tissues may exist tissue specificity, and TLR4/MyD88/NF-κB inflammatory response regulated by signal channels may have the greatest effect on retinal vessels. From this we can put forward a hypothesis: *TLR4*Asp299Gly gene polymorphism may be a susceptibility indicator of DR and the detection of this susceptibility gene can be used as a clinical diagnostic index, which may be a new target for drug therapy of DR in the future.

A number of studies have found that distribution of *TLR4*Asp299Gly site is population specificity. African populations have the highest Asp299Gly mutation rates, and then Caucasians, and the probability of mutation in Asian population, especially in Chinese Han population, is very low [39–41]. The study in Zhao et al. [42] found no genetic variation in Asp299Gly and Thr399Ile in Chinese Han populations, which is consistent with Huayin Cai’s findings [11]. Because *TLR4*Asp299Gly gene polymorphism varies widely among races, we performed a subgroup analysis of different ethnic groups in terms of Caucasian and Asian populations. The results showed that *TLR4*Asp299Gly gene polymorphism was associated with increased risk of DMI in Caucasian patients with T2DM under dominant model and allele model (dominant model OR = 1.69, 95% CI (1.22–2.35), P = 0.002; allelic model OR = 1.56, 95% CI (1.10–2.21), P = 0.01). This gene polymorphism may be a risk factor for DMI in the Caucasian population. Therefore, it may be used as a target for early prevention and treatment. But in the Asian population we found no correlation (dominant model OR = 0.85, 95% CI (0.52–1.39), P = 0.52; allelic model OR = 0.89, 95% CI (0.56–1.39), P = 0.60). This further confirmed that there are racial differences in *TLR4*Asp299Gly gene polymorphism. It's worth noting that the absence of GG genotype of *TLR4*Asp299Gly in some studies [15, 17, 20], So not all studies were included in recessive model and additive model analysis, which may have a certain impact on our final results.

Furthermore, we performed sensitivity analysis. The results showed that the corresponding OR value did not change significantly after the combination under the dominant model, which suggested that the overall results were statistically robust under this genetic model. However, the merge OR value and 95% CI (OR = 0.95, 95% CI (0.72–1.25), P = 0.70; heterogeneity: P = 0.81, I^2^ = 0) were significantly altered under the allelic model and the heterogeneity disappeared, suggesting that the heterogeneity among the meta-included studies may be attributed to these studies which are excluded by us. At the same time, we can conclude that there was no correlation between *TLR4*Asp299Gly gene polymorphism and DMI in the allelic model after sensitivity analysis.

In order to explain the results obtained better, some limitations of this meta-analysis should not be ignored. Firstly, DMI are a complex metabolic disease with multiple genetic and environmental factors. This meta analysis does not take into account the effects of other loci of *TLR4* gene or other susceptible genes polymorphisms and environmental factors. Secondly, some studies have shown that glycated albumin(GA) can be used as a predictor of diabetes, its microvascular complications and cardiovascular prognosis compared with fasting glucose(FPG) and glycated hemoglobin(HbA1c), and it would be more convincing if GA was detected in our meta-included studies[43, 44]. Thirdly, there was heterogeneity among the studies included in the dominant and allelic model analysis. The heterogeneity was not completely eliminated by subgroup analysis with different race and difference of DMI types and sensitivity analysis.

## Conclusions

In conclusion, this meta analysis showed that *TLR4*Asp299Gly gene polymorphism was associated with increased risk of DMI under dominant model, while no significant correlation was observed under allelic model, recessive model and additive model. *TLR4*Asp299Gly gene polymorphism may be considered as one of the risk factors for DMI. More importantly, our study have shown that this gene polymorphism increased the risk of DR in patients with T2DM, but not DN or diabetic neuropathy. Therefore, the detection of *TLR4*Asp299Gly can be used as a clinical diagnostic index and may be a new target for drug therapy of DR in the future.

## Data Availability

The datasets used and/or analysed during the current study are available from the corresponding author on reasonable request.

## Abbreviations

TLR4: Toll like receptor 4
MyD88: Myeloid differentiation factor 88
NF-KB: Nuclear factor-kappa B
PCR: Polymerase chain reaction
SSP-PCR: Polymerase chain reaction sequence specific primers
PCR–RFLP: PCR-restriction fragment length polymorphism
HWE: Hardy–Weinberg equilibrium
DMI: Diabetic microvascular complications
DR: Diabetic retinopathy
DN: Diabetic nephropathy
DPN: Diabetic polyneuropathy
HCY: Homocysteinemia
TnT: Troponin T
ADMA: Asymmetric dimethylarginine
GA: Glycated albumin
BMI: Body mass index
FBG: Fasting blood glucose
HbA1c: Hemoglobin A1c
TIR1: Interleukin 1
LPS: Lipopolysaccharide
HSP: Heat shock protein
HMGB1: High Mobility Group Box 1 protein
OX-LDL: Oxidized low density lipoprotein
